# Evaluating a Social-Emotional Training Program for Refugee Families and Service Providers: Pilot Study

**DOI:** 10.2196/42606

**Published:** 2023-05-05

**Authors:** Redab Al-Janaideh, Ruth Speidel, Tyler Colasante, Tina Malti

**Affiliations:** 1 Research and Assessment Centre for Leadership and Learning York Region District School Board York Region, ON Canada; 2 Centre for Child Development, Mental Health and Policy Department of Psychology University of Toronto Mississauga, ON Canada; 3 Faculty of Education Leipzig University Leipzig Germany

**Keywords:** social-emotional training, strengths-based, mental health, refugee children, refugee caregivers, service providers

## Abstract

**Background:**

Refugee children are often exposed to adversities that present a threat to their healthy development. Promoting refugee children’s social-emotional capacities may be an opportune, strengths-based avenue to nurture their resilience, coping strategies, and mental health outcomes amid these risks. Furthermore, supporting caregivers’ and service providers’ capacities to provide strengths-based care may result in more sustainable, caring environments for refugee children. However, culturally adapted initiatives that aim to promote social-emotional capacities and mental health in refugee children, caregivers, and service providers are limited.

**Objective:**

In this pilot study, we aimed to assess the feasibility and efficacy of a brief, 3-week social-emotional training program for refugee caregivers of children aged between 2 and 12 years and service providers who support refugees. This study had 3 central objectives. First, we examined whether refugee caregivers’ and service providers’ knowledge of core social-emotional concepts increased from pre- to posttraining, whether these increases were maintained 2 months later, and whether caregivers and service providers reported a high use of training-based strategies after the training. Second, we assessed if refugee caregivers reported any improvements in their children’s social-emotional capacities and mental health from pre- to posttraining and 2 months later. Finally, we evaluated whether caregivers and service providers experienced any improvements in their own mental health symptoms from pre- to posttraining and 2 months later.

**Methods:**

A total of 50 Middle Eastern refugee caregivers of children (n=26) aged between 2 and 12 years and service providers (n=24) were recruited using convenience sampling and participated in a 3-week training program. Training sessions were delivered via a web-based learning management system and involved a combination of asynchronous (video-based) and synchronous (web-based live group) sessions. The training was evaluated using an uncontrolled pre-, post-, and 2-month follow-up design. Caregivers and service providers reported their understanding of social-emotional concepts and mental health at pre-, post-, and 2 months after training and reported their use of training strategies after training. Caregivers reported their children’s social-emotional capacities and mental health through a presurvey, a series of postsurveys (after each module session and 1 week after the training), and a 2-month follow-up survey. The participants also reported their demographic information.

**Results:**

Caregivers’ and service providers’ knowledge of social-emotional concepts increased significantly from pre- to posttraining, and the service providers’ knowledge increase was sustained at the 2-month follow-up. Both caregivers and service providers reported high levels of strategy use. Furthermore, 2 markers of children’s social-emotional development (ie, emotion regulation and sadness over wrongdoing) improved after training.

**Conclusions:**

The findings highlight the potential of strengths-based, culturally adapted social-emotional initiatives to support refugee caregivers’ and service providers’ abilities to provide high-quality social-emotional care to refugee children.

## Introduction

### Background

The scale of forced migration in parts of the Middle East has reached unprecedented levels owing to increased violence and political instability [1]. For example, after 11 years of civil war, more than half of Syria’s population has been forcibly displaced, and they constitute the largest proportion of global refugees [2]. Many of these refugees are families with young children who have experienced severe pre- and postmigratory adversities (eg, violence, family separation, and discrimination), which make them vulnerable to social-emotional and mental health challenges [3,4]. Although such adversities present true risks, they do not necessarily predestine refugees to adverse outcomes. In fact, many refugees show signs of positive adjustment and some even show signs of flourishing following resettlement, which may indicate the presence of resilience or protective factors [5-7]. Although much research in this area adopts a lens of risk, there are growing calls to better understand and nurture the unique strengths of refugees [8,9]. For example, growing work shows that strengths-based factors, such as social-emotional capacities and positive early relationships, can support refugee children’s mental health and buffer against the negative impacts of adversity [10,11]. In this study, we evaluate a pilot training program designed to help refugee caregivers’ and their service providers’ promote refugee children’s social-emotional capacities and mental health, as well as to nurture the mental health of refugee caregivers and service providers themselves.

### Social-Emotional Capacities as Protective Factors in the Refugee Context

Social-emotional capacities characterize our abilities to monitor our emotions in different social settings, form caring relationships with others, and understand how we as individuals relate to the broader world around us [12]. Previous research among nonrefugee community samples showed robust associations between social-emotional capacities and positive social, behavioral, academic, and mental health outcomes in childhood and adulthood, both concurrently and longitudinally [13-18]. Extending to contexts of risk, social-emotional capacities have been identified as protective factors in children exposed to different forms of adversity, including violence (for reviews, see the articles by Fritz et al [19] and Yule et al [20]). Growing research and clinical recommendations suggest that the promotion of social-emotional capacities may be a unique opportunity for tailored intervention for refugee families and their children because of its alignment with refugees’ potential for resilience, coping skills, and optimism for the future [21,22].

Theoretical frameworks identify the following 3 core capacities of social-emotional development: emotion regulation, other-oriented social-emotional processes (eg, empathy for others), and self-oriented social-emotional processes (eg, empathy for the self; [23,24]). Importantly, these particular capacities developed rapidly during early and middle childhood, are moldable to socialization-related influences and can act as protective factors in context of adversity [25]. The current training focuses on promoting refugee caregivers’ and service providers’ knowledge of, and capacity to promote, these 3 capacities in children.

*Emotion regulation* is the ability to manage the occurrence, strength, and expression of our emotions and behaviors to achieve goals and behave appropriately in our surroundings [26,27]. Emotion regulation is an important early social-emotional process because it helps children cope with difficult emotions and situations in a variety of settings and allows them to engage in their world with greater attention, problem solving skills, and emotional maturity [28,29]. Furthermore, because emotion regulation has been identified as a key indicator of social competence, academic achievement, and mental health, it is considered an important gateway goal of early socialization [30,31]. Emotion regulation has also been identified as protective in the refugee context. For example, refugee youth who show better emotion regulation skills also tend to show better adjustment and mental health outcomes as indexed by lower internalizing, externalizing, and posttraumatic stress symptoms [11,32]. Therefore, nurturing and building upon refugee children’s emotion regulation skills may strengthen their capacity to cope amid various stressors and challenges.

*Other-oriented social-emotional processes* include those capacities that characterize how children engage with and relate to others [23]. Empathy for others is an other-oriented process that occurs when we experience a similar emotion or the same emotion as another. Sympathy refers to feelings of concern for another but is differentiated from empathy in that it does not always involve experiencing a similar or the same emotion as another [29]. Here, we use the term empathy for others to encapsulate both empathy and sympathy [29]. Empathy for others is an important social-emotional process because it enables children to interact effectively and positively in their social world. Sharing others’ emotions can help children understand when to engage in caring, prosocial behavior, and thus establish strong peer relationships [33-35]. Although empirical evidence examining other-oriented capacities in the refugee context is limited; there is an indication that empathy for others reflects a strength in this context. For example, Speidel et al [11] found that Syrian refugee youth aged 5-12 years with higher empathy for others also had lower externalizing symptoms. Furthermore, theory in the area of adversity and posttraumatic growth suggests that empathy for others may be a unique strength that characterizes resilient individuals who have experienced negative life events [36,37]. Therefore, nurturing refugee children’s other-oriented emotions, such as their empathy for others, may be an important means to support their mental health.

*Self-oriented social-emotional processes* involve how children view themselves as individuals in relation to others. Sadness over wrongdoing is a self-oriented social-emotional process that includes feelings of guilt and regret that occur when children realize that their actions may have harmed another [29]. Sadness over wrongdoing is conceptualized as a kind of emotion because it reflects an internalized understanding of, and compliance with, a guiding principle of compassion; it encourages children to repair harm caused or to avoid causing similar harm in the future [29]. Self-conscious emotions such as sadness over wrongdoing may protect against violence given that they are linked to lower aggression [18,38]. Notably, there is a distinction between healthy sadness over wrongdoing and unhealthy sadness over wrongdoing (often referred to as neurotic guilt), which may be particularly important in the refugee context [29]. Unhealthy sadness over wrongdoing occurs when a child feels disproportionate regret or shame after a real or imagined transgression that does not align with the reality. In the refugee context, this might represent feelings of survivor guilt or responsibility for others’ well-being. Some studies have indicated that unhealthy sadness over wrongdoing is associated with higher posttraumatic stress disorder symptoms in refugee youth [39]. Healthy sadness over wrongdoing involves a true understanding of and gentleness with our own limitations, thus reflecting an underlying empathy for the self [29]. Healthy sadness over wrongdoing may be protective in the refugee context because it can support children’s ability to build and maintain relationships [29]. Furthermore, because self-oriented social-emotional processes involve an underlying ability to self-reflect and understand one’s own strengths and limitations, these processes can nurture children’s capacities to engage in self-kindness and intrapersonal growth, which may also be particularly important capacities for refugee children and their care providers. In line with our strengths-based approach, we focus on healthy sadness over wrongdoing in this study.

### Caregivers and Service Providers as Socialization Agents in the Refugee Context

During the early years, social-emotional capacities are all socialized to some degree. Family- and community-based socializing agents, such as caregivers (ie, parents) and other caregiving figures (eg, educators and service providers), can serve as critical supports who positively (or negatively) influence refugee children’s early social-emotional development and mental health [40]. In the refugee context, the quality of the caregiver-child relationship is especially vital because refugee caregivers are at an elevated risk of psychological distress owing to their potential exposure to adverse experiences, which can in turn affect their capacity to engage in positive parenting behaviors [40,41]. Positive parenting behaviors, such as warmth and sensitivity, have been associated with positive child social-emotional and mental health outcomes in refugee families [42,43]. Thus, promoting caregivers’ capacities to support their own and their children’s social-emotional well-being and mental health may be especially relevant in the refugee context.

Extending socialization-based supports beyond the family level may help sustain more inclusive, caring environments that nurture refugee families’ well-being after resettlement [44]. Refugees often experience exploitation, human rights violations, and discrimination, which can lead to a general distrust of service providers and serve as barriers to accessing support [45]. Supporting service providers’ capacities to nurture positive relationships with the refugee children and families they serve, may be an important means of cultivating more inclusive, caring environments in which the strengths and needs of different communities are recognized and served. Furthermore, promoting the social-emotional capacities of service providers may increase the quality of interactions between refugee caregivers and service providers, thus facilitating refugees’ access to appropriate care. This approach may even improve mental health outcomes among service providers themselves, who tend to be at risk of secondary traumatic stress and burnout [46]. Hence, supporting both refugee caregivers and providers who serve refugee children and families may nurture change at 2 ecologically important levels of refugee children’s socialization.

### Existing Intervention and Training Efforts for Refugee Children and Families

Although trainings related to improving caregivers’ and service providers’ understanding of and ability to nurture children’s social-emotional development have shown positive effects on children’s emotional and behavioral outcomes, these initiatives often focus on typically developing children or have not been adapted to refugee populations (for reviews, see the articles by Robersten et al [47] and Stewart-Brown et al [48]). In addition, many existing efforts to support refugee children and families focus on promoting mental health and are evaluated by marking changes in deficit-based indicators, such as maladjustment, rather than assessing improvements in strengths-based indicators [49]. Other existing refugee caregiver training or intervention efforts tend to target reducing coercive or harsh caregiving behaviors (eg, [50]) or strengthening the quality of the parent-child relationship (eg, [51,52]). These existing efforts are in many ways complementary to nurturing refugee children’s social-emotional development because they support mechanisms linked to child social-emotional development and mental health (ie, caregiver warmth and emotion coaching; [53]). However, there is still a notable need for tailored interventions that specifically recognize and build upon the unique social-emotional potential of refugee children and families [54].

Similar to refugee caregiver trainings, there is a dearth of efforts promoting refugees service providers’ social-emotional knowledge. Even beyond the refugee context, gaps exist in the effective integration of social-emotional content into early care and service settings [23,55,56]. In fact, service providers often report limited training opportunities and confidence in their own abilities to support child social-emotional development in a sustainable manner [57-60]. Amid these existing gaps, there are even more prominent gaps in refugee-tailored training opportunities for service providers, with many service providers expressing challenges in building relationships and providing culturally sensitive services for refugee children [61]. To the best of our knowledge, no previous refugee-tailored caregiver or service provider trainings exist that aim to improve knowledge of social-emotional development. The current training strives to fill these gaps by supporting refugee caregivers and their service providers with a culturally adapted social-emotional training that not only promotes children’s social-emotional capacities and mental health but also supports caregivers’ and service providers’ own mental health.

### The Current Training: Supporting Prosociality and Resilience in Newcomer Transitions

The supporting prosociality and resilience in newcomer transitions (SPRINT) training was informed in part by previous social-emotional training (Speidel, R, unpublished data, February 2023). From this foundation, the training was carefully adapted based on the results of a community-based needs assessment of refugee caregivers and service providers [62]. This needs assessment corroborated evidence of pandemic-related impacts on refugees’ mental health, social and interpersonal relationships, and access to community services [62,63]. Refugee caregivers and service providers reported inadequate resources to support their own mental health and cited mental health stigma as a barrier to accessing services. Service providers further reported gaps in their training and professional development opportunities that affect their ability to understand the strengths and meet the unique needs of refugee children and families (eg, language barriers and cultural or social differences [62]). However, caregivers and service providers also highlighted supportive parenting practices, community support, and strong social and interpersonal connections among refugee children and families as notable strengths. Thus, the current training was developed to fill these identified gaps and incorporate identified strengths. The refugee adaptation process was implemented at multiple levels, such as content, language, and culture. With respect to content, the training targeted misconceptions regarding mental health in an effort to be sensitive toward mental health stigma. For example, the introductory training session clarified what topics would be introduced and their importance for overall health and well-being. Furthermore, the training adopted a strengths-based approach by encouraging caregivers to recognize and reflect upon their children’s social-emotional strengths and for service providers to reflect on and note the social-emotional strengths of the refugee children they cared for. Furthermore, given the identified strengths of community support from the needs assessment [62], the training incorporated a web-based group session component in which caregivers and service providers had the opportunity to share their lived experiences and learn from the experiences of others in their community. With respect to language accommodation, the training was delivered in Modern Standard Arabic, and refugee caregivers with reading difficulties were assisted individually. Finally, given the unique cultural and religious customs of the Middle Eastern population, we ensured that the training reflected a safe learning environment for them. For example, live sessions with participating Middle Eastern refugee caregivers were only led by women.

The training lasted for 1 month and was delivered virtually through 3 core modules, with each module delivered weekly in Arabic (for refugee caregivers) and English (for service providers). There was also a brief introductory session 1 week before the training and 2 follow-up sessions (1 week and 2 months after the training, respectively). To support flexible and interactive learning, each module included an asynchronous individual (prerecorded video) and synchronous group (web based) session. *Module 1* overviewed child social-emotional development with a focus on the following 3 core capacities in line with the extant social-emotional theory: emotion regulation, other-oriented social-emotional processes (eg, empathy for others), and self-oriented social-emotional processes (eg, sadness over wrongdoing). *Module 2* emphasized the importance of early relationships to support healthy child social-emotional development, with an empirically based focus on following 3 core caregiving processes: connecting (ie, warmth), caring (ie, sensitivity), and coaching (ie, structure, autonomy support). Finally, *Module 3* highlighted mental health and well-being and the impacts of stress on social-emotional development and mental health. This final module emphasized the importance of considering the well-being of the whole child and identified protective factors that support refugee children’s, caregivers’, and service providers’ well-being during and after stressful situations.

The asynchronous sessions included three 15-minute prerecorded videos that caregivers and service providers watched on their own time during the week. Each asynchronous session included key definitions of concepts, their developmental importance, their typical development from ages 2 to 12 years, and practical strategies to support them. After each asynchronous session, participants received activities to practice applying what they learned with their children at home or with children in their programming, ensuring that the use of strategies was well integrated into daily interactions. The synchronous sessions were 1-hour long and used the following three-part structure: (1) a group mindfulness exercise, (2) a *key points* review and group discussion of that week’s asynchronous module session, and (3) small and large group activities. The synchronous sessions emphasized reflection and practice, with participants being given the opportunity to share their experiences and actively practice applying concepts and strategies in a respectful and culturally friendly setting, which allowed them to learn from others’ diverse experiences and share creative ideas on implementing the material.

### This Study: Evaluating SPRINT

Given that very limited work exists evaluating culturally adapted trainings, we did not form a specific directional hypothesis for this pilot study. Instead, we were interested in evaluating the efficacy of the training in either direction (increase or decrease) on participants’ knowledge, mental health, and children’s mental health. This study had 3 central objectives. First, we examined whether refugee caregivers’ and service providers’ knowledge of core social-emotional concepts increased from pre- to posttraining, whether these increases were maintained after 2 months , and whether caregivers and service providers reported high use of training-based strategies after the training. Second, we assessed if refugee caregivers reported any improvements in their children’s social-emotional capacities and mental health from pre- to posttraining and after 2 months. Finally, we evaluated whether caregivers and service providers experienced any improvements in their own mental health symptoms from pre- to posttraining and after 2 months.

## Methods

### Participants

A total of 50 Middle Eastern refugee caregivers (n=26) and service providers (n=24) participated in the 3-week pilot training in fall of 2021. Participants were recruited using convenience sampling from 2 sites in Canada, the Greater Toronto and Hamilton Area in Ontario and Calgary in Alberta, using social media flyers and through word of mouth in collaboration with our community partners at the Calgary Catholic Immigration Society and the Muslim Council of Greater Hamilton. Eligibility criteria included being a resettled Syrian or Middle Eastern refugee caregiver of a child aged 2-12 years or being a service provider who works with refugee families. Service providers’ years of work experience ranged between 2 months and 33 years (mean 9.08, SD 10.10 years) in varied positions (eg, settlement workers, afterschool program coordinators, and crisis therapists). Table 1 provides detailed demographic information on refugee caregivers and service providers.

**Table 1:** Demographic information.

Variable		Refugee caregiver (n=26)	Service provider (n=24)
Age (years), mean (SD)		37.45 (7.84)	37 (12.99)
Child age (years), mean (SD)		9.52 (2.84)	N/A^a^
**Gender, n (%)**			
	Woman	23 (88)	22 (92)
	Man	0 (0)	1 (4)
	Nonbinary	0 (0)	1 (4)
	Choose not to answer	3 (12)	0 (0)
**Child (gender), n (%)**			
	Boy	10 (43)	0 (0)
	Girl	13 (57)	0 (0)
Child schooled before resettlement in Canada, n (%)		9 (35)	0 (0)
**Migration status, n (%)**			
	Migrated to Canada from another country	21 (81)	0 (0)
	Choose not to answer	5 (19)	0 (0)
Refugee camp experience, n (%)		4 (15)	0 (0)
**Population group, n (%)**			
	White	0 (0)	5 (22)
	South Asian	0 (0)	2 (9)
	Black	0 (0)	1 (4)
	Filipino	0 (0)	1 (4)
	Latin American	0 (0)	1 (4)
	Arab	24 (92)	9 (39)
	South Asian	0 (0)	1 (4)
	Korean	0 (0)	1 (4)
	Indigenous	0 (0)	1 (4)
	Italian	0 (0)	1 (4)
	Kurdish	1 (4)	0 (0)
	Choose not to answer	1 (4)	0 (0)
**Education, n (%)**			
	High school or equivalent	10 (38)	2 (8)
	Secondary or equivalent	1 (4)	0 (0)
	College or equivalent	3 (12)	2 (8)
	University degree	5 (19)	12 (50)
	Postgraduate degree	1 (4)	8 (33)
	Choose not to answer	6 (23)	0 (0)
**Employment, n (%)**			
	Full-time	1 (4)	15 (63)
	Part-time	1 (4)	6 (25)
	Unemployed	20 (74)	1 (4)
	Other	0 (0)	2 (8)
	Choose not to answer	5 (19)	0 (0)
**Income (CAD $)^b^, n (%)**			
	<$10,000	4 (15)	1 (6)
	$10,000-$49,999	8 (30)	4 (25)
	$50,000-$99,999	3 (11)	5 (31)
	≥$100,000	0 (0)	6 (38)
	Choose not to answer	12 (44)	0 (0)
**Marital status, n (%)**			
	Single	1 (4)	8 (33)
	Married	20 (77)	11 (46)
	Separated	0 (0)	3 (13)
	Divorced	1 (4)	0 (0)
	Widowed	1 (4)	1 (4)
	Choose not to answer	3 (12)	1 (4)

^a^N/A: not applicable.

^b^At the time of the study, the Canadian to US exchange rate was CAD $1 (US $0.75).

### Ethical Considerations, Informed Consent, and Participation

The study was conducted in accordance with the guidelines of the Declaration of Helsinki and was approved by the University of Toronto Research Ethics Board (39028). The caregivers and service providers provided written informed consent before participation. The consent form was available in both Arabic and English. Each participant received a CAD $50 (US $37) gift card and a confirmation of completion document as a token of appreciation for their participation. The confidentiality of participants’ identities was maintained by deidentifying all study data and assigning participants a unique ID number.

### Procedure

The training was evaluated using an uncontrolled pre-, post-, and 2-month follow-up design. To facilitate training evaluation, caregivers and service providers completed a presurvey, a series of postsurveys (after each module session and 1 week after the training), and a 2-month follow-up survey. Caregivers with multiple children were asked to choose one of their children in the age range of 2-12 years to report for the purposes of evaluation. The training was delivered virtually via a learning management system and was facilitated by a developmental psychologist with a PhD and a research assistant with experience in refugee services.

### Measures

#### Demographic Information

For the presurvey, caregivers and service providers completed a demographic questionnaire to collect information regarding age, gender, work, and immigration status in Canada. In addition, caregivers completed the Alberta Language Environment Questionnaire-4 [64] to provide information regarding the family and the participating child (ie, age, family background, length of residency in Canada, and refugee camp experience).

#### Training Evaluation Variables

##### Satisfaction With the Training

After each asynchronous and synchronous training session, caregivers and service providers rated a single item evaluating their satisfaction with the session (“What was your overall satisfaction with this training session?”). In addition, 1 week after the training, caregivers and service providers rated their satisfaction with the training in general on a single item (“What was your overall satisfaction with the training?”). These items were developed by the second, third, and final authors and were rated on a 10-point scale (1=*very dissatisfied*, 4=*somewhat dissatisfied*, 7=*somewhat satisfied,* 10=*very satisfied*).

##### Use of Training Strategies

A week after the training was completed, caregivers and service providers reported their use of training-based strategies (eg, mindfulness and emotion coaching) using a single item; “*To what extent do you use any of the strategies from the training*?.” The item was rated on a 10-point scale (1: *not at all*, 4: *sometimes*, 7: *often*, 10: *very often*) and was developed by the second, third, and final authors.

##### Knowledge of Training Content

At pretraining, after each asynchronous and synchronous training session, and at the 2-month follow-up, caregivers and service providers rated their understanding of core social-emotional training-based concepts using 18 items. The items assessing knowledge of the training content were developed based on adult education principles, emphasizing the importance of adult learning opportunities informing knowledge, competencies, and reflective practice [65]. In total, 9 of the items evaluated knowledge of key developmental concepts that were the central focus of the training (“I understand what emotion regulation is and how it develops”) and the remaining 9 items were designed to assess knowledge of applying key competency elements that were the central focus of the training (eg, “I know how to support children’s emotion regulation*”*)*.* These items were developed by the second, third, and final authors. Individual scores across the 18 items were averaged to form the pretraining, posttraining (responses of each asynchronous and synchronous training session were averaged to create a single posttraining score), and 2-month follow-up scores. The internal consistency of the composite scores was high at each time point (Cronbach α_caregiver_=.93, .89, and .97, respectively; Cronbach α_service provider_=.95, .91, and .96, respectively).

#### Caregiver and Service Provider Well-being Variables

##### Anxiety Symptoms

At pretraining, 1-week posttraining, and the 2-month follow-up, caregivers and service providers reported their anxiety symptoms over the previous 2 weeks using 2 items from the Generalized Anxiety Disorder-2 ([66]; “Feeling nervous, anxious, or on edge,” “Not being able to stop or control worrying”). The items were rated on a 4-point scale (0: *not at all*, 1: *several days*, 2: *over half the days*, and 3: *nearly every day*) and were averaged to form a composite anxiety score. Bivariate correlations suggested that the 2 items held together adequately as a single construct at each time point (refugee caregiver: Pearson *r*=0.78, 0.66, and 0.79; service provider: Pearson *r*=0.52, 0.53, and 0.45).

##### Depressive Symptoms

At pretraining, 1-week posttraining, and the 2-month follow-up, caregivers and service providers reported their depressive symptoms over the previous 2 weeks using 2 items from the Patient Health questionnaire-2 ([67]; “Little interest or pleasure in doing things,” “Feeling down, depressed, or helpless”). Items were rated on a 4-point scale (0: *not at all*, 1: *several days*, 2: *over half the days*, and 3: *nearly every day*) and were averaged to form a composite depression score. Bivariate correlations suggested that the 2 items held together adequately as a single construct over time (refugee caregiver: Pearson *r*=0.66, 0.48, and 0.40; service provider: Pearson *r*=0.71, 0.70, and 0.59, respectively).

#### Child Variables

##### Child Social-Emotional Capacities

At pretraining, 1-week posttraining, and the 2-month follow-up, caregivers reported their child’s emotion regulation, empathy for others, and sadness over wrongdoing using the Social-Emotional Responding Task [23,68]. These capacities were assessed because they map onto each of the following 3 core social-emotional capacities highlighted in the training: emotion regulation, other-oriented social-emotional capacities, and self-oriented social-emotional capacities. Caregivers completed 12-items assessing emotion regulation (eg, “My child calmly deals with what is making them mad,” Cronbach α=.93, .89, and .93), 4 items assessing empathy for others (eg, “My child feels bad for other children who are sad,” Cronbach α=.81, .72, and .71), and 4 items assessing sadness over wrongdoing (eg, “When my child does something that makes another child feel sad, they feel sad,” Cronbach α=.62, .76, and .63). Items were rated on a 4-point scale (0: *not at all*, 1: *sometimes true*, 2: *often true*, and 3: *almost always true*) and were averaged to form emotion regulation, empathy, and sadness over wrongdoing scores.

##### Child Mental Health Challenges

At pretraining, 1-week posttraining, and the 2-month follow-up, caregivers reported their children’s mental health challenges using the Arabic version of the Strengths and Difficulties Questionnaire [69,70], a widely used and validated measure of Arabic samples [71,72]. Specifically, caregivers completed 20 items tapping into children’s internalizing and externalizing symptoms, with each item rated on a 3-point scale (0: *not true*, 1: *somewhat true*, and 2: *certainly true*). The items were averaged to form a total mental health challenge variable, with higher scores reflecting higher mental health challenges (Cronbach α=.68, .92, and .88).

### Data Analytic Plan

To address our research questions, we conducted 7 repeated measures ANOVAs (ie, separate ANOVAs to assess change in each outcome variable of interest measured at pre-, post-, and 2 months after training: caregiver and service provider knowledge, child emotion regulation, child empathy for others, child sadness over wrongdoing, child mental health challenges, caregiver and service provider anxiety, and caregiver and service provider depression) using SPSS 28 (IBM Corp). To assess differences in caregivers’ versus service providers’ changes in knowledge, anxiety, and depressive symptoms, we included group status (0: *service provider* and 1: *caregiver*) as a between-subjects factor in these repeated measures ANOVAs. The Greenhouse-Geisser results were reported when Mauchly test of sphericity was statistically significant. When significant univariate effects emerged, follow-up pairwise comparisons were performed using Bonferroni corrections. To evaluate our research question regarding the prevalence of posttraining strategy use, we used a descriptive analysis of the means, SDs, and ranges of caregiver and service provider reports on this variable.

### Missing Data Analysis

On average, the participants completed 64% (32/50) of the training evaluation surveys. To evaluate patterns of missingness, Little test of missing completely at random was conducted for all study variables and was nonsignificant (*χ*^2^_144_=160.9; *P*=.15) suggesting that the missing data did not violate the assumption of missing completely at random. To handle missing data, we used multiple imputations (n=10) in SPSS 28 to maximize the power of the analysis. The 10 imputed data sets were averaged to generate single final scores for each variable (ie, complete data for analyses).

## Results

### Overview

The descriptive statistics for all the measures are presented in Table 2. With respect to training satisfaction, 96% (24/25) of caregivers reported being somewhat satisfied (ie, 7 out of 10 on our scale) to very satisfied (ie, 10 out of 10) after each training session, as well as 1 week after the training was completed (with half, 13/26, 50%, reporting being very satisfied). A total of 96% (23/24) of service providers reported being somewhat satisfied to very satisfied after each session, and 1 week after the training was completed (6/24, 25% reported being very satisfied).

**Table 2:** Descriptive statistics.^a^

Variables			Refugee caregiver					Service provider							Full sample			
			Mean (SD)		Range		Mean (SD)			Range			Mean (SD)				Range	
**Participant training variables**																		
	Knowledge (before)	7.91 (1.13)		5.33-9.94		8.28 (1.16)			5.67-10.00			8.09 (1.15)				5.33-10.00		
	Knowledge (after)	8.71 (0.91)		5.39-10.00		8.97 (0.45)			8.50-10.00			9.03 (0.58)				7.00-10.00		
	Knowledge (2-month)	8.51 (1.38)		6.00-10.00		8.92 (0.78)			7.00-10.00			8.74 (1.03)				5.67-10.00		
	Use of strategies (after)	8.34 (1.15)		5.00-10.00		7.8 0 (1.06)			5.00-10.00			8.08 (1.13)				5.00-10.00		
**Participant well-being variables**																		
	Anxiety (before)	0.78 (0.74)		0.00-3.00		0.79 (0.76)			0.00-2.50			0.79 (0.74)				0.00-3.00		
	Anxiety (2-month)	0.61 (0.61)		0.00-3.00		0.71 (0.46)			0.00-2.00			0.66 (0.62)				0.00-3.00		
	Depression (after)	1.01 (0.79)		0.00-3.00		0.68 (0.71)			0.00-2.50			0.85 (0.76)				0.00-3.00		
	Depression (2-month)	1.00 (0.79)		0.00-2.50		0.45 (0.43)			0.00-1.39			0.74 (0.69)				0.00-2.50		
**Child variables**								N/A^b^			N/A			N/A				N/A
	Emotion regulation (before)	1.39 (0.61)		0.33-2.92														
	Emotion regulation (2-month)	1.64 (0.57)		0.42-3.00														
	Empathy (before)	1.99 (0.65)		1.00-3.00														
	Empathy (2-month)	2.24 (0.50)		1.00-3.00														
	Sadness over wrongdoing (before)	1.76 (0.49)		0.99-2.75														
	Sadness over wrongdoing (2-month)	2.12 (0.48)		1.00-3.00														
	Mental health challenges (before)	11.79 (4.01)		4.00-20.00														
	Mental health challenges (after)	15.45 (6.80)		2.00-32.00														
	Mental health challenges (2-month)	12.06 (6.96)		1.00-25.45														

^a^The actual allowable ranges for each variable are as follows: knowledge (0-10), use of strategies (1-10), depression (0-3), anxiety (0-3), child emotion regulation (0-3), child empathy (0-3), child sadness over wrongdoing (0-3), and child mental health challenges (0-2).

^b^N/A: not applicable.

### Research Question 1: Changes in Caregivers’ and Service Providers’ Knowledge of Core Social-Emotional Concepts and Use of Training Strategies

**Figure 1:**
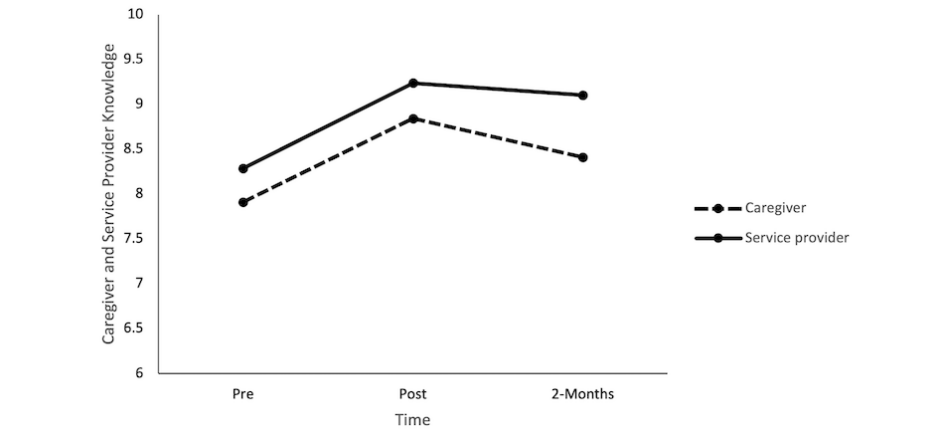
Caregiver and service provider knowledge at pretraining, posttraining, and the 2-month follow-up.

The repeated measures ANOVA results revealed there was a statistically significant effect of time, *F*_2,96_=22.46, *P*<.001, η^2^=0.31, although this effect was qualified by a within-between interaction, *F*_1,48_=5.68, *P*=.02, η^2^=0.10, indicating that there was a difference in caregivers’ versus service providers’ changes in knowledge over time (Figure 1). Service providers’ knowledge significantly increased from pretraining to posttraining (mean difference 0.94, SE 0.22, *P*<.001) and from pretraining to the 2-month follow-up (mean difference 0.81, SE 0.22, *P*=.003) but did not change from posttraining to the 2-month follow-up (mean difference 0.13, SE 0.16, *P*>.99). The latter effect indicated that increases in service provider knowledge were sustained 2 months after the training was completed. Among refugee caregivers, knowledge significantly increased from pretraining to posttraining (mean difference 0.93, SE 0.21, *P*<.001). However, caregiver knowledge did not increase significantly from pretraining to the 2-month follow-up (mean difference 0.50, SE 0.22, *P*=.08), and there was a significant decline in knowledge from posttraining to 2-month follow-up (mean difference 0.43, SE 0.15, *P*=.02). Thus, the initial increases in refugee caregivers’ knowledge upon finishing the training partially subsided 2 months after training.

The descriptive analysis of strategy use indicated that caregivers and service providers reported high strategy use after training (caregiver: mean 8.34, SD 1.15; Range 5-10; service provider mean 7.80, SD 1.06; Range 5-10), as the mean scores were between the anchor points of *often* (7) and *very often* (10), and 74% (37/50) of respondents reported using training-based strategies *often* or more. Caregivers and service providers reported similar strategy use, t_48_=−1.72, *P*=.09.

### Research Question 2: Changes in Child Social-Emotional Capacities and Mental Health

**Figure 2:**
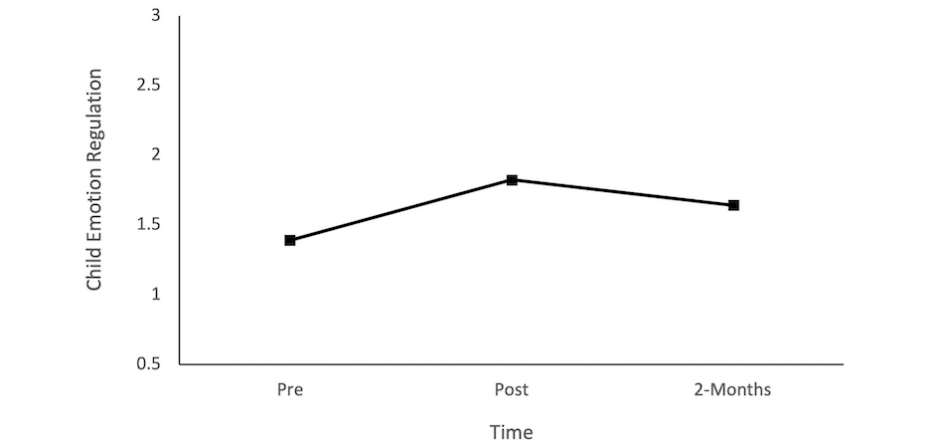
Child emotion regulation at pretraining, posttraining, and the 2-month follow-up.

Repeated measures ANOVA results revealed a statistically significant change in child’s emotion regulation between at least 2 time points, *F*_1.67,41.83_=7.87, *P=*.002, η^2^=0.24. Follow-up pairwise comparisons revealed a significant increase in child emotion regulation from pre- to posttraining (mean difference 0.43, SE 0.12, *P=*.008). However, the changes from pretraining to the 2-month follow-up (mean difference 0.25, SE 0.11, *P*=.09) and from posttraining to the 2-month follow-up were not statistically significant (mean difference 0.18, SE 0.08, *P*=.14; Figure 2), suggesting that the initial increases in child emotion regulation were not fully sustained by the 2-month follow-up.

**Figure 3:**
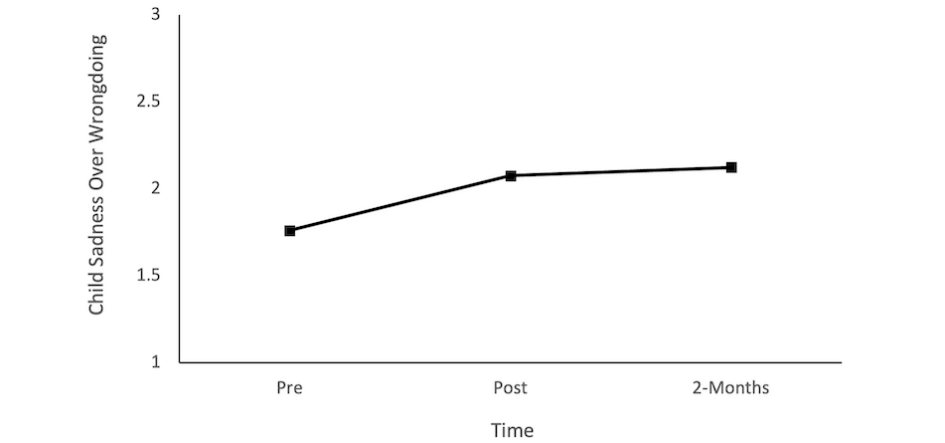
Child sadness over wrongdoing at pretraining, posttraining, and the 2-month follow-up.

Finally, with respect to children’s sadness over wrongdoing, repeated measures ANOVA results revealed an overarching effect of time, *F*_1.99,49.80_=7.99, *P*<.001, η^2^=0.24. Follow-up pairwise comparison results indicated a significant increase in children’s sadness over wrongdoing from pre- to posttraining (mean difference 0.31, SE 0.09, *P=*.01) and from pretraining to the 2-month follow-up (mean difference 0.36, SE 0.10, *P=*.004), with no significant change in sadness over wrongdoing from posttraining to the 2-month follow-up (mean difference 0.04, SE 0.09, *P*>.99, Figure 3). This pattern of results suggested that children’s sadness over wrongdoing increased at posttraining and maintained that level for 2 months.

Results of repeated measures ANOVA revealed no statistically significant changes in children’s empathy, *F*_1.91,47.77_=2.08, *P=*.14, η^2^=0.07, or mental health challenges over time, *F*_1.89,47.39_=2.84, *P=*.07, η^2^=0.10.

### Research Question 3: Changes in Caregiver and Service Provider Depressive and Anxiety Symptoms

**Figure 4:**
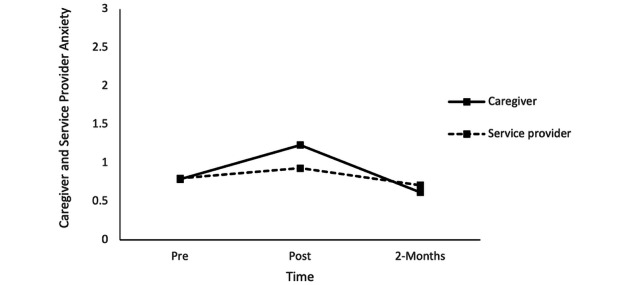
Caregiver and service provider anxiety symptoms at pretraining, posttraining, and the 2-month follow-up.

With respect to anxiety symptoms, repeated measures ANOVA results revealed a statistically significant effect of time, *F*_1.94,93.31_=6.27, *P=*.003, η^2^=0.11. Furthermore, changes in anxiety symptoms were similar across time among caregivers and service providers (*F*_1,48_=0.243, *P*=.62, η^2^=0.00). There was no significant change in anxiety symptoms from pre- to posttraining (mean difference 0.29, SE 0.13, *P=*.09) or from pretraining to the 2-month follow-up (mean difference 0.12, SE 0.11, *P=*.78). However, there was a statistically significant decrease in anxiety symptoms from posttraining to the 2-month follow-up (mean difference 0.41, SE 0.12, *P*=.003; Figure 4).

**Figure 5:**
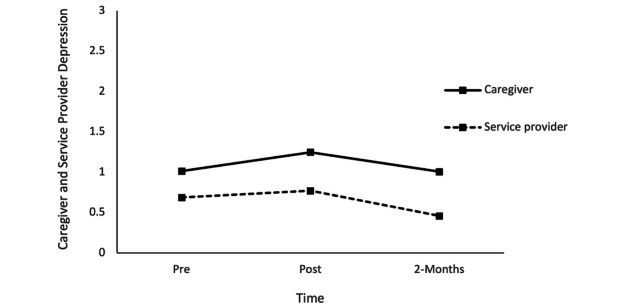
Caregiver and service provider depression symptoms at pretraining, posttraining, and the 2-month follow-up.

With respect to depressive symptoms, repeated measures ANOVA results revealed that there was no statistically significant change in depressive symptoms over time, *F*_1.88,90.55_=2.55, *P=*.09, η^2^=0.05. However, there were significant differences in depressive symptoms among caregivers and service providers, *F*_1,48_=13.94, *P*<.001, η^2^=0.22, as service providers were significantly lower in depressive symptoms relative to caregivers at posttraining (mean difference 0.47, SE 0.15, *P*=.004) and the 2-month follow-up (mean difference 0.54, SE 0.18, *P*=.004), but not at pretraining (mean difference 0.32, SE 0.21, *P*=.13; Figure 5).

## Discussion

### Key Findings and Implications

The goal of this study was to evaluate the feasibility and effectiveness of a pilot training for refugee caregivers of children aged 2-12 years and service providers who support refugees. Preliminary results indicated that caregivers and service providers reported increases in their knowledge of core social-emotional concepts and high strategy use after the training was completed. Furthermore, caregivers reported improvements in 2 indices of child social-emotional capacities, that is, child emotion regulation and sadness over wrongdoing. We address our results in detail in the subsequent section.

Both caregivers and service providers reported significant increases in their knowledge of social-emotional concepts from pre- to posttraining, indicating that the training was successful in improving caregivers’ and service providers’ theory- and research-based knowledge of critical social-emotional developmental constructs. However, there were differences between caregivers and service providers regarding the continuation of this knowledge gain at the 2-month follow-up. Service providers’ knowledge increase was sustained at the 2-month follow-up, whereas that of caregivers showed a decline back to pretraining levels. These results highlight the evidence of training sustainability among service providers. This result may suggest that the training showed compatibility with service providers’ professional learning needs and their capacities to translate learning into services and care. Importantly, the current training fills a gap in service providers’ training opportunities because many educators and practitioners working with refugee families report gaps in their self-efficacy related to building relationships and identifying and nurturing the unique social-emotional strengths and needs of the refugee children and families they serve [62]. Notably, the SPRINT training uses a blended learning structure in which participants engaged with the core knowledge-based components of each module in a self-paced manner according to their own schedules and needs, and these modules were complemented by weekly web-based sessions that emphasized group discussion and reflection. The success of this blended learning mode is further reflected in service providers’ high satisfaction level with the training (an average of 96% reported being somewhat satisfied to very satisfied with the training). This corroborates previous research on the effectiveness of blended learning, especially when combined with evidence-based pedagogical practices [73,74].

We did not observe evidence of the same sustained knowledge gains among refugee caregivers. Instead, refugee caregivers reported initial increases in their knowledge at posttraining, which subsided during the 2-month follow-up. Notably, similar to service providers, refugee caregivers also reported high satisfaction with the training and high strategy use at posttraining, which suggests that many caregivers showed high engagement with the training content. It is possible that caregivers experienced more challenges translating information from the training into their daily interactions and behaviors with their children, which may have stymied the sustainability of their knowledge gains 2 months after training. It is also possible that the cultural gap between refugee caregivers and their children, as discussed during open web-based discussions, made it more difficult for them to sustain the applicability of strategies over time. Refugee caregivers and their children may benefit from trainings in the future, placing a greater focus on open communication. It is also possible that the hybrid training delivery or brief length of training was insufficient to maintain sustained learning among refugee caregivers. More targeted or individualized training, perhaps including refugee children in the fold and booster sessions, may have further supported caregiver knowledge.

The results from this study showed that 2 of the 3 child social-emotional indicators (emotion regulation and sadness over wrongdoing) improved from pre- to posttraining. Furthermore, significant improvements were sustained 2 months after the training for children’s sadness over wrongdoing. Improvements in child emotion regulation and sadness over wrongdoing underscore the potential utility of brief social-emotional trainings directed at refugee families and their children, as well as their potential compatibility with other resilience- and family-based training [75]. This finding also supports the ecological framework theory [76] in the sense that promoting children’s relationships within their microsystems (eg, parents) may be beneficial for their social-emotional development. The current findings are particularly promising as emotion regulation and self-oriented social-emotional processes can facilitate children’s coping and interpersonal development amid adversities [21,22]. In the refugee context, family-based trainings may be particularly efficacious as they aim to support the broader family system and not just children or caregivers individually. Indeed, Middle Eastern refugee families stress extended family and interpersonal support as critical strengths during and beyond their resettlement [62]. From a family systems theory perspective, a family-wide approach may mitigate the intergenerational effects of pre- and postmigration traumas that often impact refugee children and their caregivers [77].

There were no significant parent-reported changes in children’s mental health challenges. It is possible that improvements in children’s mental health symptoms may emerge further downstream through changes in other factors, such as improved parenting behaviors, improved parent-child relationship quality, and improved child social-emotional capacities [78]. It is also possible that brief and largely strengths-based social-emotional trainings may not be sufficient to support the oftentimes complex mental health needs of refugee children. Such trainings may need to be supplemented with more targeted and individualized mental health supports for children who present with more severe psychopathologies or well-being–related challenges. With respect to the impact of the training on participants’ mental health, there were no changes in caregiver and service provider depression, and there were no changes in their anxiety from pre- to posttraining or from pretraining to the 2-month follow-up. However, caregivers and service providers experienced significant decreases in their anxiety symptoms from posttraining to the 2-month follow-up. Although it is important to interpret this effect with caution, it could suggest a delayed effect of the training on caregiver and service provider anxiety. Notably, refugee caregivers reported higher levels of depressive symptoms compared with service providers at posttraining and at the 2-month follow-up. Given the vulnerable nature of the refugee population, as well as the possible traumatic experiences and challenges associated with their resettlement, this finding is consistent with previous research indicating that refugees tend to have higher levels of depressive symptoms than other populations (for a review, see the article by Lindert et al [79]). These findings are particularly important because parental well-being has been tied to family well-being (eg, children and caregivers), and trainings aimed at improving parental well-being have been found to promote positive parenting behaviors [80].

This study has several limitations. First, and perhaps most importantly, the lack of a control group prevented us from drawing firm conclusions regarding the efficacy of the training. Given that the training was a pilot, we thought it would be more ethical to include all participants in the experimental group, and given the project’s short timeline, having a delayed group was also not an option. Future studies should include a control group to allow for more precise conclusions. Another limitation of this study is the relatively small sample size, which, albeit appropriate for a pilot study, limits the power and generalizability of our results. Future training evaluations should strive for larger sample size. Moreover, despite efforts to encourage attendance, participant attendance was understandably low given that the training took place during COVID-19 and frequent transition between in-person and web-based school and work settings caused disruptions to participants’ schedules. Future initiatives should consider devising and implementing novel strategies to encourage participants to attend virtually.

In conclusion, SPRINT training provided overall evidence of its effectiveness and feasibility. Refugee caregivers and service providers reported high satisfaction with the training, suggesting that brief strengths-based training can be beneficial for refugee populations. The current findings highlight the need for more tailored and culturally adapted training, and pave the way for more research and evaluation of social-emotional strengths among refugee families and children.

## Abbreviations

SPRINT: supporting prosociality and resilience in newcomer transitions

## Data Availability

The data sets generated and analyzed during this study are not publicly available because the research team was not granted ethical approval to do so but are available from the corresponding author on reasonable request.
